# Melatonin alleviates septic ARDS by inhibiting NCOA4-mediated ferritinophagy in alveolar macrophages

**DOI:** 10.1038/s41420-024-01991-8

**Published:** 2024-05-24

**Authors:** Wenting Xu, Yutong Wu, Sheng Wang, Song Hu, Yu Wang, Wenyu Zhou, Yuanli Chen, Quanfu Li, Lina Zhu, Hao Yang, Xin Lv

**Affiliations:** 1 Department of Anesthesiology, Shanghai Pulmonary Hospital, School of Medicine, Tongji University, Shanghai, 200433 People’s Republic of China; 2 Anhui Medical University, Hefei, Anhui 236000 People’s Republic of China; 3 The First Affiliated Hospital, Zhejiang University School of Medicine, Hangzhou, 310000 People’s Republic of China

**Keywords:** Medical research, Pathogenesis

## Abstract

Ferroptosis is a novel form of programmed cell death which can exacerbate lung injury in septic acute respiratory distress syndrome (ARDS). Alveolar macrophages, crucial innate immune cells, play a pivotal role in the pathogenesis of ARDS. Ferritinophagy is a process of ferritin degradation mediated by nuclear receptor coactivator 4 (NCOA4) which releases large amounts of iron ions thus promoting ferroptosis. Recent evidence revealed that inhibiting macrophage ferroptosis can effectively attenuate pulmonary inflammatory injury. Melatonin (MT), an endogenous neurohormone, has antioxidant and anti-inflammatory effects and can reduce septic ARDS. However, it is not clear whether MT’s pulmonary protective effect is related to the inhibition of macrophage ferritinophagy. Our in vitro experiments demonstrated that MT decreased intracellular malondialdehyde (MDA), Fe^2+^, and lipid peroxidation levels, increased glutathione (GSH) levels and cell proliferation, and upregulated glutathione peroxidase 4 (GPX4) and ferritin heavy chain 1 (FTH1) protein levels in LPS-treated macrophages. Mechanistically, the antiferroptotic effect of MT on LPS-treated macrophages was significantly compromised by the overexpression of NCOA4. Our in vivo experiments revealed that MT alleviated the protein expression of NCOA4 and FTH1 in the alveolar macrophages of septic mice. Furthermore, MT improved lipid peroxidation and mitigated damage in alveolar macrophages and lung tissue, ultimately increasing the survival rates of septic mice. These findings indicate that MT can inhibit ferroptosis in an NCOA4-mediated ferritinophagy manner, thereby ameliorating septic ARDS.

## Introduction

Acute respiratory distress syndrome (ARDS) is a critical clinical syndrome with high morbidity and mortality [1–3] that is characterized by diffuse alveolar injury and increased alveolar capillary permeability [4]. There are multiple pathogeneses of ARDS, including sepsis, multiple emergency transfusions, aspiration, and pulmonary contusion and so on, among which sepsis is one of the most common causes, accounting for approximately 43% of ARDS cases [5]. Currently, there is no effective clinical treatment for ARDS, and management primarily involves supportive therapies such as antibiotics, hormones, and mechanical ventilation [6, 7]. As a result, many patients with severe sepsis and ARDS face challenges in their recovery, with an overall in-hospital mortality rate of up to 40% [8–10]. Finding an effective treatment option is of the utmost importance for ARDS patients.

Alveolar macrophages are the main intrinsic immune cells in the lungs and play crucial roles in the immune process, serving as the first line of defense against invasion by exogenous pathogens such as bacteria, fungi and toxic contaminants [11]. Macrophages are involved in regulating iron homeostasis, and intracellular iron accumulation can induce M1-type macrophage polarization. M1-type macrophages release inflammatory factors and initiate inflammation [12]. Studies have shown a link between ferroptosis and macrophages, and ferroptotic macrophages release various inflammatory cytokines, including IL-1β, IL-6, and TNF-α [13].

Ferroptosis is a novel form of programmed cell death that is distinct from apoptosis, pyroptosis, necrosis, or autophagy and is mainly caused by excessive intracellular Fe^2+^ accumulation. Excessive accumulation of Fe^2+^ generates large amounts of highly toxic reactive oxygen species (ROS) through the Fenton and Haber-Weiss reactions, ultimately leading to cellular ferroptosis and lipid peroxidation damage [14]. Key proteins involved in cellular ferroptosis include glutathione peroxidase 4 (GPX4), ferritin heavy chain 1 (FTH1), and nuclear receptor coactivator 4 (NCOA4) [15]. GPX4 participates in the antioxidant process, FTH1 is an important component of ferritin, and NCOA4 is a coactivator of multiple nuclear receptors that localize in the nucleus [15]. NCOA4 binds to FTH1 to facilitate the delivery of ferritin to autophagosomes. The fusion of autophagosomes with lysosomes leads to ferritin degradation and iron release, which is known as ferritinophagy [16]. Ferritinophagy has been implicated in the development and progression of various pathological processes and diseases, such as diabetes, neurodegeneration, and cardiovascular diseases [17–19]. Inhibiting ferritinophagy has been shown to effectively mitigate macrophage ferroptosis and alleviate inflammation, and can sustain erythropoiesis [20, 21]. However, the role of ferritinophagy in septic ARDS remains unclear. The present study focuses on ferritinophagy in ARDS and its potential mechanism.

Melatonin (MT) is a hormone that is primarily secreted by the pineal gland that exerts multiple regulatory effects, such as regulating sleep rhythm, circadian circulation, immune defense, and anti-inflammatory, and antioxidant effects [22–25]. Numerous studies have shown that MT is a potent endogenous antioxidant that can indirectly stimulate the expression of antioxidant enzymes, such as superoxide dismutase (SOD) and glutathione peroxidase (GPx), thereby enhancing the antioxidant capacity of tissues [26–30]. Moreover, MT possesses chelating properties and can reduce toxicity caused by metal accumulation [31]. Recent studies have confirmed the therapeutic effect of MT in acute lung injury [32, 33]. Additionally, MT has been proven to inhibit ferroptosis in some diseases, such as acute kidney injury and traumatic brain injury [34, 35]. A recent study reported that MT improves ferritinophagy in acute cardiotoxicity [19]. However, whether MT can alleviate ARDS by inhibiting macrophage ferritinophagy is still unclear.

In the present study, we stimulated murine macrophages (RAW264.7 cells) with lipopolysaccharide (LPS) and intraperitoneally injected mice with LPS to establish a septic ARDS model. We investigated whether MT could alleviate septic ARDS by inhibiting ferritinophagy in vitro and in vivo, and explored the underlying mechanism. Our findings may provide novel methods and strategies for the treatment of septic ARDS.

## Results

### LPS induces ferroptosis in macrophages

**Fig. 1:**
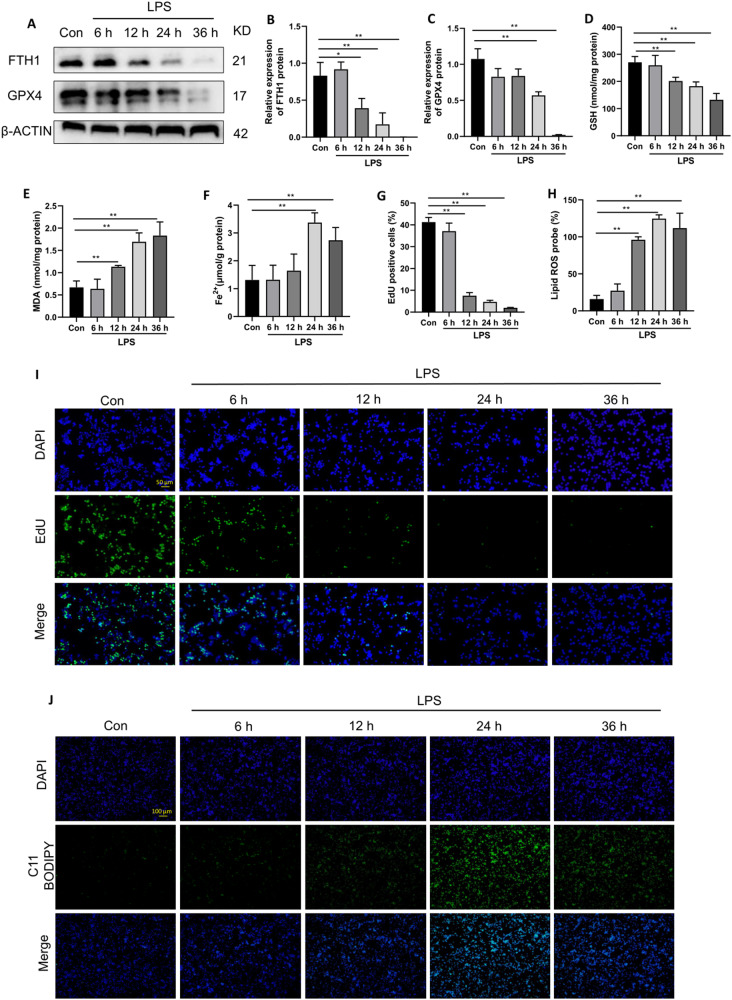
Ferroptosis in RAW264.7 cells is activated with LPS (1 μg/ml) at different time points. **A**–**C** The FTH1 and GPX4 protein levels were detected by western blot. Intracellular GSH (**D**), MDA (**E**), and Fe^2+^ (**F**) contents were detected by corresponding assay kits. Cell proliferation detected by the EdU assay kit was observed under a fluorescence microscope (**I**) and statistics as shown in (**G**). Lipid peroxidation levels detected by the C11 BODIPY assay kit were observed under a fluorescence microscope (**J**) and statistics as shown in (**H**). Data are expressed as mean ± SD (n = 6), *p < 0.05, **p < 0.01 indicate significant differences from each group, NS indicates no significance.

To confirm the induction of ferroptosis by LPS in macrophages and determine the optimal time point, RAW 264.7 cells (a murine macrophage cell line) were treated with 1 μg/ml LPS and collected at different time points after LPS stimulation. The results demonstrated a time-dependent decrease in the protein expression of FTH1 and GPX4, which were lowest at 24 and 36 h (Fig. 1A–C). Additionally, the levels of GSH (a reductive agent for antioxidation), the lipid peroxidation product MDA and intracellular Fe^2+^ were assessed by corresponding assay kits. LPS stimulation significantly reduced GSH levels (Fig. 1D) and increased MDA (Fig. 1E) and intracellular Fe^2+^ (Fig. 1F) levels, and the most significant effects were observed at 24 or 36 h. Moreover, cell proliferation was diminished in a time-dependent manner and was evident after 12 h (Fig. 1G, I). Similarly, intracellular lipid peroxidation levels were increased after LPS stimulation and peaked at 24 h (Fig. 1H, J). These results confirmed that LPS could induce ferroptosis in RAW264.7 cells, and the most pronounced effects were observed at 24 h. Therefore, 24 h was chosen as the time point for the subsequent in vitro experiments.

### MT can effectively inhibit LPS-induced ferroptosis in macrophages

**Fig. 2:**
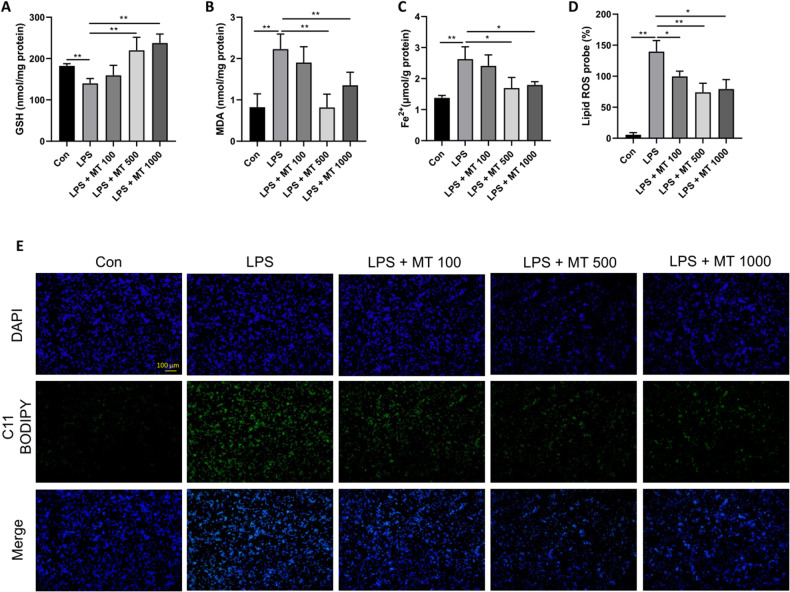
Different doses of MT inhibit LPS-induced ferroptosis in RAW264.7 cells. Intracellular GSH (**A**), MDA (**B**), and Fe^2+^ (**C**) contents were detected by corresponding assay kits. Lipid peroxidation levels detected by the C11 BODIPY assay kit were observed under a fluorescence microscope (**E**) and statistics as shown in (**D**). Data are expressed as mean ± SD (n = 6), *p < 0.05, **p < 0.01 indicate significant differences from each group, NS indicates no significance.

MT is an important antioxidant and anti-inflammatory agent that plays a crucial role in decreasing oxidative stress and inhibiting proinflammatory cytokine overproduction in lung tissues [36]. However, the relationship between ferroptosis (iron-dependent lipid peroxidation) and MT in septic ARDS is still unknown. To determine an appropriate and effective dose of MT to inhibit macrophage ferroptosis, different doses of MT (100, 500, and 1000 μM) were administered to LPS-stimulated RAW264.7 cells. The results showed that LPS stimulation significantly reduced GSH levels, and MT treatment reversed this effect in a dose-dependent manner, increasing GSH levels in macrophages (Fig. 2A). Conversely, MDA (Fig. 2B) and intracellular Fe^2+^ levels (Fig. 2C) decreased after MT supplementation (500 and 1000 μM) compared with those in the LPS group. Additionally, MT reduced lipid peroxidation levels in LPS-treated RAW264.7 cells, and the most significant effect was observed in response to 500 μM (Fig. 2D, E). In summary, 500 μM MT was selected as the interventional dose with RAW264.7 cells in the following in vitro experiments.

**Fig. 3:**
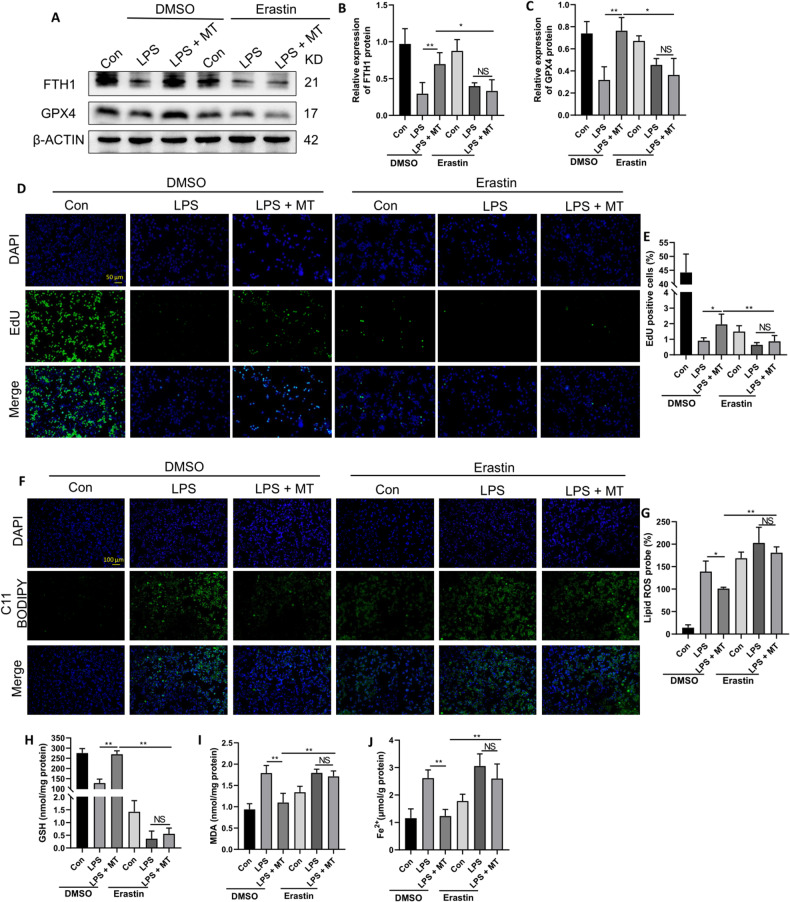
MT markedly alleviates ferroptosis in macrophages after LPS stimulation. **A**–**C** The FTH1 and GPX4 protein levels were detected by western blot. Cell proliferation detected by the EdU assay kit was observed under a fluorescence microscope (**D**) and statistics as shown in (**E**). Lipid peroxidation levels detected by the C11 BODIPY assay kit were observed under a fluorescence microscope (**F**) and statistics as shown in (**G**). Intracellular GSH (**H**), MDA (**I**), and Fe^2+^ (**J**) contents were detected by corresponding assay kits. Data are expressed as mean ± SD (n = 6), * *p* < 0.05, **p < 0.01 indicate significant differences from each group, NS indicates no significance.

To further confirm that MT can mitigate ferroptosis in LPS-treated macrophages, RAW264.7 cells were pretreated with the ferroptosis agonist erastin. As shown in Fig. 3A–C, MT treatment upregulated the protein expression of FTH1 and GPX4 in LPS-treated RAW264.7 cells, but this protective effect was reduced by erastin. Furthermore, erastin pretreatment decreased cell proliferation (Fig. 3D, E) and cellular GSH levels (Fig. 3H) in the LPS + MT group. Moreover, the inhibitory effects of MT on lipid peroxidation levels (Fig. 3F, G) and intracellular MDA and Fe^2+^ levels in LPS-treated RAW264.7 cells were effectively alleviated after pretreatment with erastin (Fig. 3I, J). These results demonstrated that MT could inhibit ferroptosis in LPS-treated macrophages.

### MT suppresses LPS-induced ferroptosis in macrophages via melatonin receptors

**Fig. 4:**
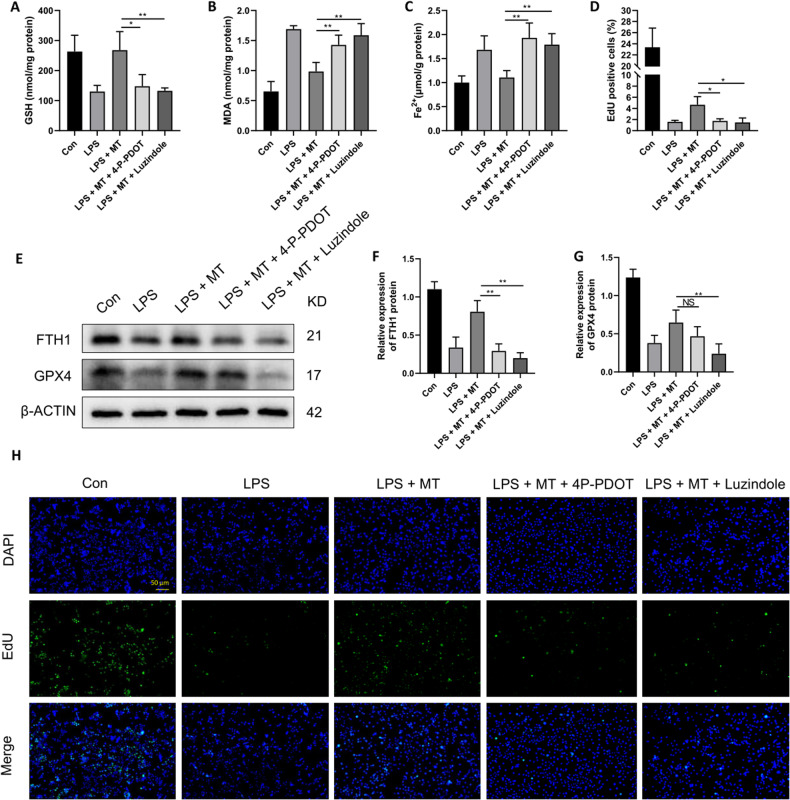
MT inhibits RAW264.7 cell ferroptosis via MT2. Intracellular GSH (**A**), MDA (**B**), and Fe^2+^ (**C**) contents were detected by corresponding assay kits. Cell proliferation detected by the EdU assay kit was observed under a fluorescence microscope (**H**) and statistics as shown in (**D**). **E**–**G** The FTH1 and GPX4 protein levels were detected by western blot. Data are expressed as mean ± SD (n = 6), *p < 0.05, **p < 0.01 indicate significant differences from each group, NS indicates no significance.

It has been discovered that MT exerts its organ protective effects mainly through its membrane receptors 1 (MT1) and/or MT2 [37]. To further investigate the mechanism underlying the antiferroptotic effects of MT on LPS-stimulated macrophages, melatonin receptor (MTR) antagonists (4-P-PDOT and luzindole) were used. 4-P-PDOT is an MT2 specific antagonist, and luzindole is a nonselective MTR antagonist. We found that both 4-P-PDOT and luzindole could effectively decrease the inhibitory effect of MT on GSH, MDA and intracellular Fe^2+^ levels in LPS-stimulated RAW264.7 cells, and luzindole had a stronger inhibitory effect (Fig. 4A–C). Furthermore, cell proliferation was inhibited by 4-P-PDOT or luzindole individually compared with that in the LPS + MT group (Fig. 4D, H). Moreover, FTH1 protein expression was downregulated by both 4-P-PDOT and luzindole, but GPX4 protein expression was only downregulated by luzindole, compared with that in the LPS + MT group (Fig. 4E–G). These results suggested that MT exerts its antiferroptotic effect through MT2. However, whether MT1 demonstrated a significant impact remains to be further explored.

**Fig. 5:**
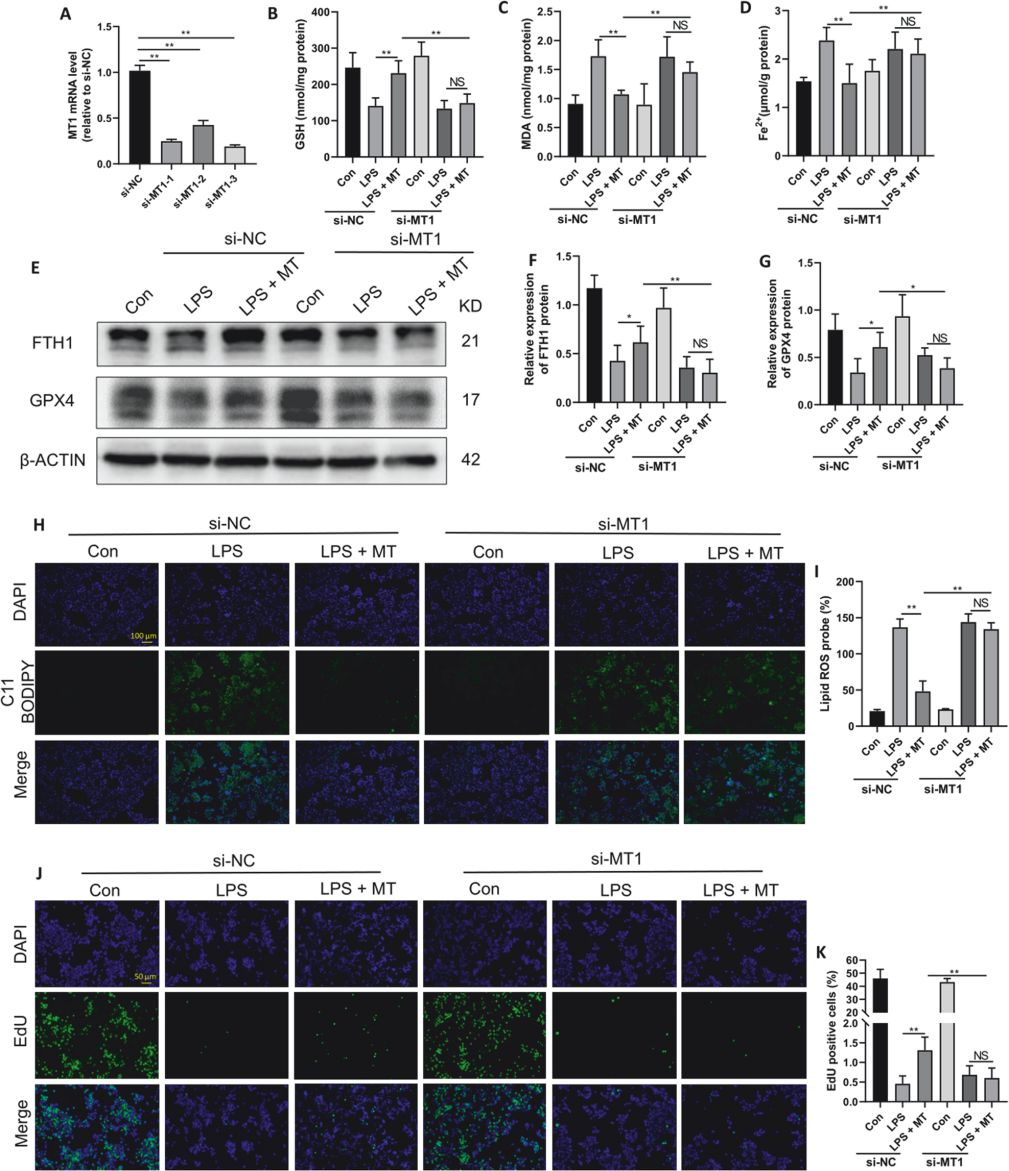
MT suppresses LPS-induced ferroptosis in RAW264.7 cells via MT1. **A** The mRNA expression levels of MT1 after knockdown of MT1 receptor in RAW264.7 cells with siRNA. Intracellular GSH (**B**), MDA (**C**), and Fe^2+^ (**D**) contents were detected by corresponding assay kits. **E**–**G** The FTH1 and GPX4 protein levels were detected by western blot. Lipid peroxidation levels detected by the C11 BODIPY assay kit were observed under a fluorescence microscope (**H**) and statistics as shown in (**I**). Cell proliferation detected by the EdU assay kit was observed under a fluorescence microscope (**J**) and statistics as shown in (**K**). Data are expressed as mean ± SD (n = 6), *p < 0.05, **p < 0.01 indicate significant differences from each group, NS indicates no significance.

Because there is a lack of MT1-specific antagonists, we used a small interfering RNA (siRNA) to specifically inhibit MT1 expression in RAW264.7 cells. The qPCR results showed the reduced mRNA expression of MT1 in RAW264.7 cells that were transfected with MT1-specific siRNA (si-MT1), and the inhibitory effect was most obvious in response to si-MT1-3 (Fig. 5A). Therefore, si-MT1-3 was selected for the following experiments.

As shown in Fig. 5B–D, the antiferroptotic effect of MT on GSH, MDA and intracellular Fe^2+^ levels in LPS-treated RAW264.7 cells was markedly weakened when MT1 was knocked down. Furthermore, the antiferroptotic effect of MT on FTH1 and GPX4 protein expression in LPS-treated RAW264.7 cells was significantly weakened after transfection with si-MT1 (Fig. 5E–G). Subsequently, cell proliferation and lipid peroxidation levels in LPS-treated RAW264.7 cells were examined. As shown in Fig. 5H, I, MT treatment significantly reduced cell lipid peroxidation levels in LPS-treated RAW264.7 cells, and this effect was decreased after MT1 knockdown. Conversely, cell proliferation (Fig. 5J, K) was increased by MT and effectively decreased by MT1 knockdown. These results indicate that MT can alleviate LPS-induced ferroptosis in RAW264.7 cells through MT1.

### MT alleviates LPS-induced ferroptosis in macrophages by inhibiting ferritinophagy

Ferritinophagy is a process in which ferric ions are released as free iron from ferritin, and this process is mediated by NCOA4, a selective cargo receptor for autophagic turnover of ferritin (ferritinophagy) [38, 39]. Our previous results demonstrated that MT could alleviate the accumulation of Fe^2+^ and decrease the expression of ferritinophagy-associated protein FTH1 in LPS-treated RAW264.7 cells. Therefore, we hypothesized that MT could alleviate ferritinophagy in LPS-treated macrophages.

**Fig. 6:**
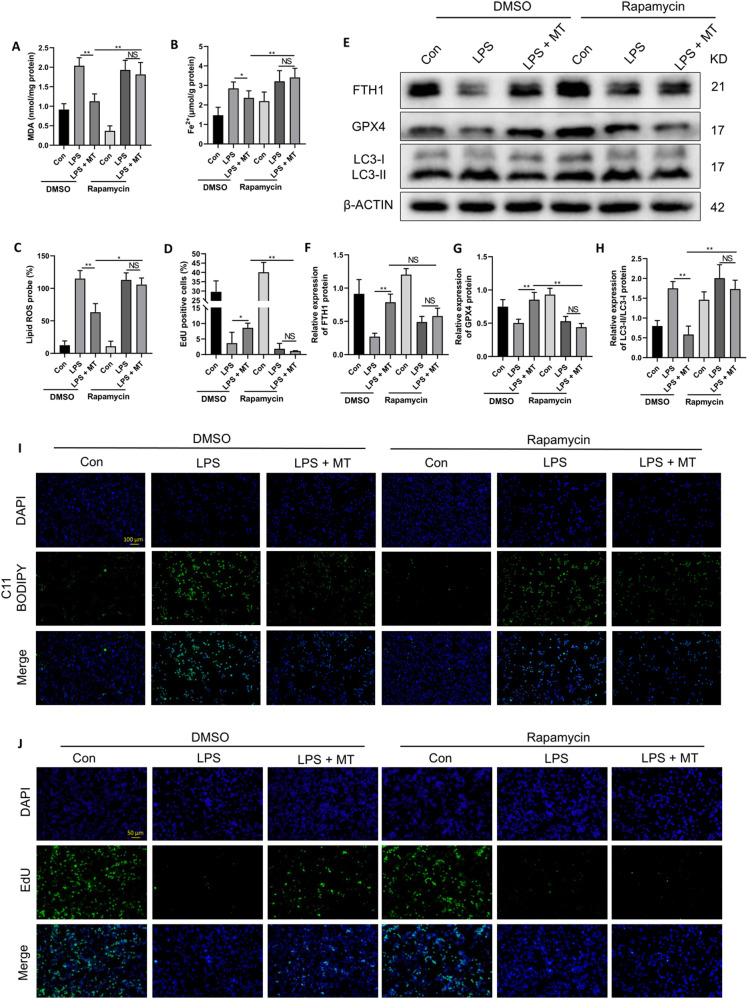
MT alleviates LPS-induced ferroptosis in RAW264.7 cells by inhibiting ferritinophagy. Intracellular MDA (**A**), and Fe^2+^ (**B**) contents were detected by corresponding assay kits. Lipid peroxidation levels detected by the C11 BODIPY assay kit were observed under a fluorescence microscope (**I**) and statistics as shown in (**C**). Cell proliferation detected by the EdU assay kit was observed under a fluorescence microscope (**J)** and statistics as shown in (**D**). **E**–**H** The FTH1, GPX4 and LC3 protein levels were detected by western blotting. Data are expressed as mean ± SD (n = 6), *p < 0.05, **p < 0.01 indicate significant differences from each group, NS indicates no significance.

To confirm this hypothesis, we activated ferritinophagy in RAW264.7 cells with the specific inducer rapamycin [40, 41]. The antiferroptotic effect of MT on intracellular MDA and Fe^2+^ levels in LPS-stimulated RAW264.7 cells was significantly attenuated by rapamycin pretreatment (Fig. 6A, B). Similarly, the MT-induced suppression of lipid peroxidation (Fig. 6C, I) and promotion of cell proliferation (Fig. 6D, J) in LPS-stimulated RAW264.7 cells were significantly attenuated by rapamycin treatment. Furthermore, the MT-induced upregulation of FHT1 and GPX4 protein expression in LPS-treated RAW264.7 cells were blocked by rapamycin administration (Fig. 6E–G). LC3 is a key protein involved in ferritinophagy, and a high LC3 II/I ratio indicates the occurrence of ferritinophagy. As shown in Fig. 6E, H, the ratio of LC3 II/I in RAW264.7 cells was upregulated after LPS stimulation but was decreased after MT treatment. In contrast, the MT-induced inhibition of the LC3 II/I ratio in LPS-treated RAW264.7 cells was attenuated by rapamycin treatment. Based on these results, MT exerts an antiferroptotic effect on LPS-treated macrophages by mitigating ferritinophagy.

### MT attenuates mitochondrial damage in LPS-treated macrophages

Important features of ferritinophagy are also reflected in the abnormal changes in mitochondrial membrane potential and mitochondrial morphology [42]. To investigate the impact of MT on mitochondrial membrane potential, we used the JC-1 mitochondrial assay kit and observed the cells under fluorescence microscopy. The red/green fluorescence ratio in RAW264.7 cells was diminished after LPS treatment, suggesting a reduction of mitochondrial membrane potential, which was restored after MT treatment. Interestingly, pretreatment with erastin and rapamycin failed to restore the mitochondrial membrane potential in RAW264.7 cells after treatment with MT (Supplementary 1A, B). Cells undergoing ferritinophagy are also characterized by mitochondrial morphological alterations, including mitochondrial swelling or wrinkling, increased membrane density, reduced or absent mitochondrial cristae, and breakage of the outer mitochondrial membrane at the ultrastructural level [43]. As shown in Supplementary 1C, LPS induced these typical features in RAW264.7 cells, including marked mitochondria swelling, vacuoles in mitochondria, almost invisible mitochondrial cristae, and even outer membrane rupture in some mitochondria. However, these morphological changes were alleviated after treatment with MT. Similarly, cells that were pretreated with erastin and rapamycin exhibited significant abnormal morphological changes compared to those in the LPS + MT group. These mitochondrial alterations further confirm that MT alleviates ferritinophagy in macrophages.

### MT alleviates LPS-induced ferroptosis in macrophages by inhibiting NCOA4-mediated ferritinophagy

**Fig. 7:**
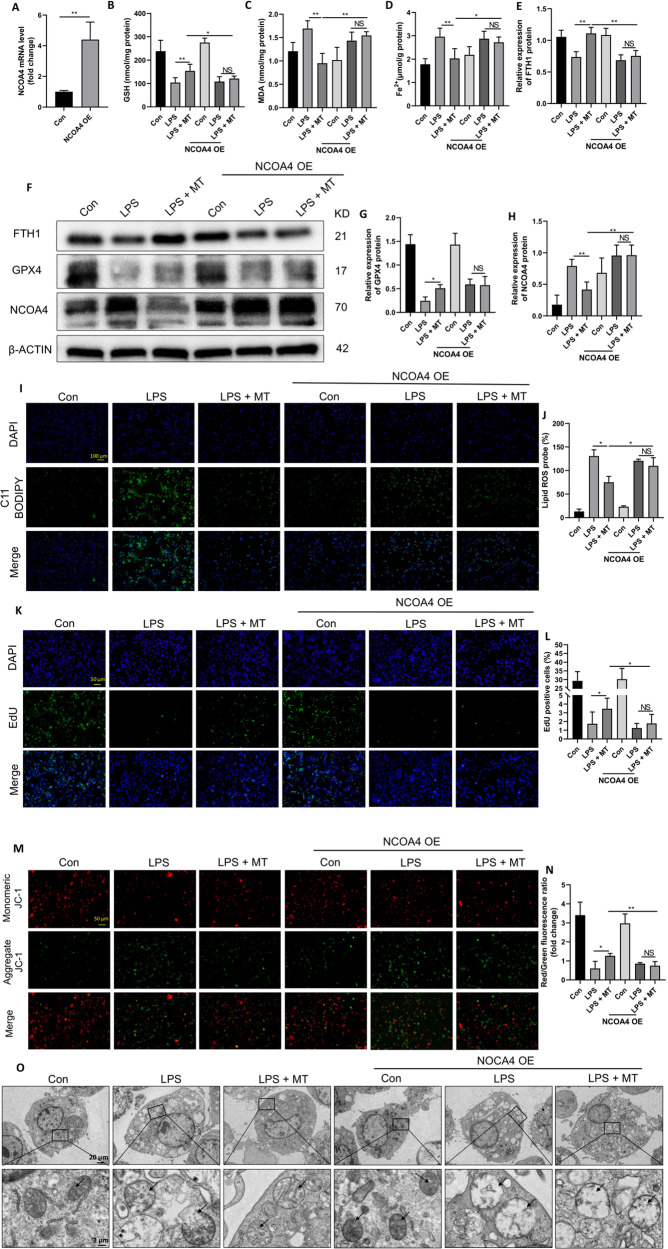
MT protects RAW264.7 cells from LPS-induced ferroptosis by inhibiting NCOA4-mediated ferritinophagy. **A** The transfection efficiency of lentivirus was detected by qPCR. Intracellular GSH (**B**), MDA (**C**), and Fe^2+^ (**D**) contents were detected by corresponding assay kits. **E**–**H** The FTH1, GPX4 and NCOA4 protein levels were detected by western blotting. Lipid peroxidation levels detected by the C11 BODIPY assay kit were observed under a fluorescence microscope (**I**) and statistics as shown in (**J**). Cell proliferation detected by the EdU assay kit was observed under a fluorescence microscope (**K**) and statistics as shown in (**L**). Mitochondrial membrane potential changes detected by JC-1 mitochondrial assay kit was observed (**M**) under fluorescence microscope and statistics as shown in (**N**). **O** Mitochondrial morphological changes under electron microscopy (The arrows mark mitochondria). Data are expressed as mean ± SD (n = 6), *p < 0.05, **p < 0.01 indicate significant differences from each group, NS indicates no significance.

NCOA4 is of great importance in ferritinophagy by binding to FTH1 and delivering ferritin to the lysosome. To investigate the role of NCOA4 in MT-mediated ferritinophagy inhibition, we overexpressed NCOA4 (NCOA4 OE) in RAW264.7 cells using a lentivirus and confirmed its overexpression by qPCR analysis (Fig. 7A). The antiferroptotic effect of MT on the intracellular levels of GSH, MDA and Fe^2+^ in LPS-treated RAW264.7 cells was significantly attenuated in NCOA4-OE cells (Fig. 7B–D). Similarly, western blot analysis revealed that NCOA4 overexpression effectively inhibited the MT-induced upregulation of FTH1 and GPX4 protein expression in LPS-treated RAW264.7 cells (Fig. 7E–H). Fluorescent staining showed that MT-induced suppression of cellular lipid peroxidation levels (Fig. 7I, J) and proliferation (Fig. 7K, L) in LPS-treated RAW264.7 cells were diminished by NCOA4 overexpression. In addition, mitochondrial membrane potential was increased by MT in LPS-treated RAW264.7 cells and was also effectively decreased after NCOA4 overexpression (Fig. 7M, N). Electron microscopy showed abnormal mitochondrial morphological alterations, such as mitochondrial swelling, intramitochondrial vacuoles, mitochondrial cristae rupture, and mitochondrial outer membrane rupture caused by LPS, and these effects were not significantly alleviated by MT in NCOA4-OE cells (Fig. 7O). These findings suggest that the inhibitory effects of MT on ferritinophagy in LPS-stimulated macrophages may be mediated by NCOA4.

### MT attenuates septic ARDS by inhibiting ferritinophagy in alveolar macrophages in vivo

**Fig. 8:**
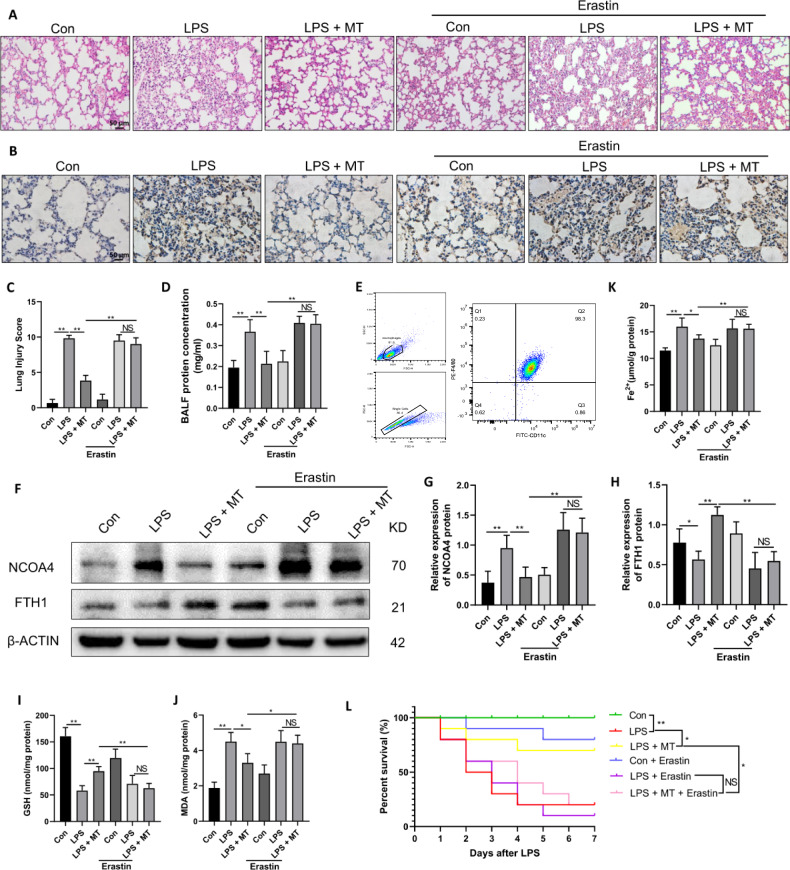
MT alleviates septic ARDS by inhibiting ferritinophagy of alveolar macrophages in mice. **A** The representative HE staining of lung tissue sections. **B** 4-HNE immunohistochemistry of mouse lung tissues. **C** The lung injury score analysis. **D** The protein concentration in BALF. **E** Alveolar macrophages were detected by flow cytometry with F4/80 and CD11c. **F**–**H** The NCOA4 and FTH1 protein levels were detected by western blot. The contents of GSH (**I**), MDA (**J**) and Fe^2+^ (**K**) in alveolar macrophages of mice. **L** The survival rate of mice. Data are expressed as mean ± SD (n = 6), *p < 0.05, **p < 0.01 indicate significant differences from each group, NS indicates no significance.

To further verify the therapeutic effect of MT on septic ARDS in vivo, a septic ARDS model was established by intraperitoneal injection of LPS in mice, followed by MT treatment 30 min later. Erastin (15 mg/kg) was intraperitoneally injected three times in advance at 12-h intervals. As expected, LPS caused pulmonary hemorrhage, interstitial edema and thickening of the alveolar wall, as observed by HE staining, and these pathological changes were attenuated by MT treatment. However, the effect of MT was attenuated in the Erastin-pretreated group (Fig. 8A). The lung injury score also supported the antiferroptotic effect of MT on septic ARDS, which was suppressed after Erastin intervention (Fig. 8C). Additionally, the level of 4-HNE, which is a marker of lipid peroxidation, was increased by LPS in lung tissues of mice and decreased after MT treatment, and it remained high in the Erastin + LPS + MT group (Fig. 8B). Furthermore, the protein levels in BALF were significantly elevated in ARDS mice but were reduced after MT treatment. However, erastin attenuated the therapeutic effect of MT (Fig. 8D).

Next, alveolar macrophages were extracted from BALF and identified by flow cytometry (Fig. 8E). The extracted alveolar macrophages are used for the following examinations. Western blot analysis revealed that MT significantly downregulated NCOA4 and upregulated FTH1 expression at the protein level in the alveolar macrophages of LPS-treated mice, and the effect of MT was greatly weakened by erastin treatment (Fig. 8F–H). Consistently, MT treatment increased GSH levels and decreased MDA levels in the alveolar macrophages in LPS-treated mice, which were both attenuated by erastin (Fig. 8I, J). The accumulation of Fe^2+^ in the alveolar macrophages of LPS-treated mice was reduced by MT treatment, but elevated by erastin compared with that in the LPS + MT group (Fig. 8K). Finally, we verified the role of MT in improving the survival of ARDS mice. The results showed that the survival rate of ARDS mice was improved by MT but remained low in erastin pretreated group (Fig. 8L). These results confirmed that MT could alleviate septic ARDS by inhibiting AM ferritinophagy in vivo.

## Discussion

The present study yielded several important findings. First, MT effectively protected RAW264.7 cells from LPS-induced ferroptosis. This protective effect was primarily mediated by the activation of MT1 and MT2 receptors. Additionally, MT exerted its antiferroptotic effect by inhibiting ferritinophagy in RAW264.7 cells. Mechanistically, the inhibitory effect of MT on ferroptosis in these cells was attributed to its ability to suppress the NCOA4-mediated ferritinophagy pathway. Importantly, in an in vivo model of septic ARDS, MT could alleviate the condition by inhibiting NCOA4-mediated ferritinophagy in alveolar macrophages. These findings emphasize the crucial role of iron overload and ferritinophagy in alveolar macrophages in the development of ARDS, suggesting that MT could be a promising therapeutic option to effectively prevent these processes via the NCOA4 pathway (Supplementary 2).

ARDS is a severe condition characterized by diffuse alveolar injury, inflammation, and alveolar hemorrhage. Due to intractable hypoxemia and the progressive exacerbation of respiratory failure, ARDS is widely discussed in the field of respiratory disease and critical care [44]. Alveolar macrophages, as pivotal immune cells in the lungs, play a significant role in the development of ARDS through diverse functions that include engulfing pathogens and debris (phagocytosis), presenting antigens to other immune cells, providing defense against infections, and regulating the body’s immune response (immunomodulation) [45–48]. The accumulation of iron stores, particularly in M1-polarized macrophages, suggests the presence of intracellular iron overload and potential ferritinophagy in macrophages during ARDS [49]. Therefore, exploring the regulation of ferritinophagy in macrophages as a therapeutic target for ARDS is of great interest. Our study demonstrated the occurrence of ferroptosis in LPS-stimulated macrophages, and there were significant alterations in iron accumulation, lipid peroxidation, and mitochondrial morphology.

MT, a hormone predominantly secreted by the pineal gland, has been demonstrated to have diverse regulatory effects and therapeutic potential in various conditions [22–25]. Clinically, it can be administered orally or by injection to exert therapeutic effects, such as treating sleep disorders, relieving anxiety and depression, preventing and treating cancer, and treating organ damage [50]. Previous studies have reported the protective effects of MT on different organs by inhibiting ferroptosis and reducing oxidative stress, which protects against cerebral ischemia-reperfusion injury, hepatitis cirrhosis, kidney injury, heart disease, and lung injury [50]. Previous studies have shown that MT has a significant effect on immune cell function and can treat macrophage-related diseases [51]. In the context of lung injury, MT has been shown to inhibit ferroptosis in lung epithelial cells alleviate ARDS [52]. However, its role in regulating macrophage ferroptosis and its potential therapeutic effect in ARDS remain unknown. In our study, we found that MT effectively upregulated the protein expression of FTH1 and GPX4, increased the level of GSH, and decreased the levels of MDA and Fe^2+^ in LPS-stimulated macrophages. Notably, the therapeutic effect of MT on ferroptosis was significantly diminished after pretreatment with erastin, a ferroptosis agonist that inhibits System xc- [53]. This finding suggests that MT can alleviate LPS-induced macrophage damage by inhibiting ferroptosis, thereby ameliorating septic ARDS. MT interacts with three distinct receptors: MT receptor 1 (MT1), MT2, and MT3. Of these, MT1 and MT2 are transmembrane G protein-coupled receptors and the main pathways through which MT exerts its biological effects [54]. MT1 plays a crucial role in the body’s internal clock system, influencing physiological processes that follow a daily rhythm and associated with regulating mood stability, pain perception [55]. On the other hand, MT2 receptors have been implicated in the development and progression of type 2 diabetes. They are believed to affect glucose metabolism and insulin sensitivity, thus playing a role in the pathogenesis of this metabolic disorder [56]. Moreover, MT2 has also been identified as an independent predictor for survival in individuals with non-small cell lung cancer (NSCLC), suggesting its potential involvement in cancer prognosis and possibly treatment response [57]. Our findings indicate that the inhibitory effect of MT on ferroptosis is mediated mainly through MT1 and MT2, as demonstrated by the use of receptor antagonists and siRNA-mediated downregulation of MT1 expression. However, further studies are needed to elucidate the specific receptors involved in MT-mediated inhibition of ferroptosis, and there may be variations depending on the specific cellular context.

Ferritinophagy is a specialized form of autophagy that targets ferritin for degradation within autophagosomes, resulting in the release of substantial quantities of iron ions. This process is intricately linked to various critical cellular processes such as growth, proliferation, differentiation, programmed cell death (apoptosis), and cancer development [16]. In our study, we observed that MT alleviated the accumulation of Fe^2+^ and FTH1 protein expression in LPS-stimulated macrophages, suggesting that MT exerts its pulmonary protective effects by inhibiting ferritinophagy. This is consistent with previous reports on the protective effect of MT by improving of ferritinophagy in acute cardiotoxicity [19]. In general, ferritin uptake is meticulously controlled by an intricate network of iron-responsive proteins to maintain intracellular Fe^2+^ balance and associated physiological functions [16]. While ferritinophagy has been implicated in certain ferritin deposition diseases and iron metabolism-related disorders, its role in septic ARDS remains unexplored [58]. Our study revealed the occurrence of ferritinophagy in LPS-stimulated macrophages, which was attenuated by MT treatment. Importantly, the inhibitory effect of MT on ferritinophagy was significantly weakened after treatment with rapamycin, a known inducer of autophagy. These findings highlight the role of MT in alleviating ARDS by inhibiting macrophage ferritinophagy, providing a novel therapeutic approach and shedding light on a previously unexplored mechanism by which MT regulates ferroptosis.

NCOA4, which is a widely expressed intracellular protein, plays a crucial role in ferritinophagy by binding to FTH1 and facilitating its delivery to autophagosomes, resulting in the excessive release of Fe^2+^. Our study demonstrated that the therapeutic effect of MT on ferritinophagy was significantly attenuated in LPS-stimulated macrophages overexpressing NCOA4, indicating that MT alleviates macrophage ferroptosis by inhibiting NCOA4-mediated ferritinophagy.

To summarize, our study provides evidence that MT protects macrophages from LPS-induced ferroptosis and alleviates septic ARDS by inhibiting NCOA4-mediated ferritinophagy. However, further investigations are required to validate these findings in animal models of ARDS. The therapeutic potential of MT in ARDS treatment warrants deeper exploration and validation.

## Conclusion

In summary, we explored the underlying mechanisms by which MT alleviates septic ARDS. The present study demonstrated that ferritinophagy occurs in alveolar macrophages during septic ARDS and that MT can alleviate ARDS by inhibiting NCOA4-mediated ferritinophagy in alveolar macrophages, providing a new avenue for exploring the mechanism by which MT alleviates septic ARDS.

## Methods

### Ethics statement

All experiments and surgical procedures described in the present study were conducted in compliance with the ethical guidelines and regulations set forth by the Animal Care and Use Committee of the Tongji University School of Medicine. The study also adhered to the recommendations outlined in the ‘Guide for the Care and Use of Laboratory Animals’ published by the National Institutes of Health and followed the relevant sections of the ARRIVE guidelines.

### Reagents

Dulbecco’s modified Eagle’s medium (DMEM), fetal bovine serum (FBS), penicillin/streptomycin, and phosphate-buffered saline (PBS) were purchased from Gibco (Grand Island, NY, USA). Antibodies against β-Actin, NCOA4, FTH1, LC3, and GPX4, as well as HRP Goat Anti-Rabbit Ig G (H + L), were obtained from ABclonal (Wuhan, China). LPS, MT, 4-P-PDOT, luzindole were purchased from Sigma-Aldrich (St. Louis, MO, USA). Erastin and rapamycin were purchased from Selleck (Houston, Texas, USA). Bicinchoninic acid (BCA) protein assay kit was purchased from Beyotime Biotechnology (Shanghai, China).

### Culture and LPS treatment of RAW264.7 cells

The RAW264.7 macrophage cell line was sourced from the American Type Culture Collection (ATCC), located in Manassas, VA, USA. These cells were cultivated in Dulbecco’s Modified Eagle Medium (DMEM, Gibco brand) which was fortified with 10% fetal bovine serum (Gibco) and an additional 1% penicillin/streptomycin antibiotic blend (Gibco). The culture conditions maintained were at a steady temperature of 37°C under a humidified atmosphere containing 5% CO_2_. RAW264.7 cells were seeded onto 10 cm cell culture dishes (Corning Inc., New York, USA) and the medium was refreshed every two days to ensure optimal growth. Cell passage was carried out once the cells achieved approximately 90% confluence. This process involved discarding the used culture medium, followed by rinsing the adherent cells twice with PBS. Subsequently, the cells were detached and re-suspended in fresh medium at a ratio of 1:3 for further propagation. In subsequent experiments, RAW264.7 cells were treated with 1 μg/ml LPS (L3024, Sigma, MO, USA) for 24 h as described in our previous publication [59]. Erastin (10 μM) (Selleck, USA), rapamycin (20 μM) (Selleck), 4-P-PDOT (10 μM) (Sigma) and luzindole (10 μM) (Sigma) were treated 3 h before LPS stimulation. MT (500 μM) (Sigma) was treated 1 h after LPS stimulation. All cells were verified to be free of mycoplasma.

### Cell proliferation determination

The EdU Cell Proliferation Image Kit (CellorLab, China) was used to measure cell proliferation of RAW264.7 cells when they were inoculated into 6-well plates and stimulated with different drugs or the same volume of DMSO accordingly. After 24 h of LPS treatment, cells were treated and stained following the manufacturer’s instructions and stained with DAPI. Cell proliferation was determined by a fluorescence microscope.

### RNA extraction and real-time quantitative PCR (RT-qPCR)

Total RNA was extracted from RAW264.7 cells or alveolar macrophages using Trizol reagent (Invitrogen, Calif). Complementary DNAs (cDNAs) were synthesized using 5 × PrimeScript RT Master Mix (Vazyme, Jiangsu, China) following the manufacturer’s instructions. RT-qPCR was performed on a Light Cycler 480 detection system (Roche, Rotkreuz, Switzerland) by the 2 × color SYBR Green qPCR Master Mix (Vazyme, Jiangsu, China). β-Actin was used as a reference gene, and the primers were designed using Primer 5.0 software, listed in Table 1. The mRNA expression levels were calculated using the 2 -ΔΔCt method.

**Table 1:** Primer sequences used for reverse transcription real-time quantitative PCR assays.

Gene primer sequence		
mRNA	Primers	Sequences (5′-3′)
β-Actin	Forward	AGTGTGACGTTGACATCCGT
	Reverse	GCAGCTCAGTAACAGTCCGC
mNCOA4α	Forward	GAGGTGTGGCTCAATGAACAGGTC
	Reverse	CACTGGATGCTGACTTCTGCTCTG
siMT1	Forward	GAAGAAGCAGAUAAGAUUAdTdT
	Reverse	UAAUCUUAUCUGCUUCUUCdTdT

### Western blot analysis

Total protein was extracted from RAW264.7 cells or alveolar macrophages according to published methods [60]. The membranes were subjected to an overnight incubation process at a controlled temperature of 4 °C, during which they were treated with primary antibodies. These antibodies were carefully diluted to a ratio of 1:1000 and included NCOA4 (Abclonal), GPX4 (Abclonal), FTH1 (ABclonal), LC3 (ABclonal) or β-Actin (ABclonal). The membranes were then incubated with secondary antibodies (diluted 1:1000, ABclonal, China) at room temperature for 1 h. Signals were detected using an advanced enhanced chemiluminescence system manufactured by Thermo Scientific, and relative protein expression was finally quantified with ImageJ software.

### Detection of lipid ROS

The suitably treated cells were incubated with 50 μmol C11 BODIPY 581/591 fluorescence probe (Thermo, USA) for 30 min in a humidified incubator (at 37 °C, 5% CO_2_). After washing twice with PBS, the cells were fixed using 4% paraformaldehyde solution to preserve cellular structures, stained with DAPI, then excited by 488 nm and 565 nm lasers respectively with an inverted fluorescence microscope to collect relevant fluorescence signal images.

### Measurement of ferroptosis-related markers

Levels of intracellular Fe^2+^, the lipid peroxidation metabolite malondialdehyde (MDA) and the reductant glutathione (GSH) were measured in RAW264.7 cells or alveolar macrophages using the Iron assay kit (Nanjing Jiancheng Bioengineering Institute), the MDA assay kit (Nanjing Jiancheng Bioengineering Institute), and the GSH assay kit (Nanjing Jiancheng Bioengineering Institute) respectively, following the manufacturer’s instructions.

### Transmission electron microscopy (TEM)

After 24 h of LPS treatment, the cells were collected and fixed with 2.5% glutaraldehyde for 30 min at room temperature in the dark. The fixed cells were then dehydrated and embedded in epoxy resin, and ultrathin sections were prepared for examination under a transmission electron microscope.

### siRNA transfection

MT1-targeting siRNAs (siMT1) or non-targeting control siRNAs (siNC) were synthesized by Genomeditech (Shanghai, China). The detailed sequences of these siRNAs are provided in Table 1 for reference. In the experimental protocol, RAW264.7 cells that were in their logarithmic growth phase were seeded into 24-well plates. These cells were then transiently transfected with either siMT1 or siNC using Lipofectamine 3000 (Invitrogen, Carlsbad, CA, USA). Cells were harvested 48 h after transfection, and used for the subsequent experiments.

### Mitochondrial membrane potential assay

Mitochondrial membrane potential alterations were assessed using the JC-1 Assay Kit (Beyotime, China). The experimental procedure involved the following steps: RAW264.7 cells were seeded in 12-well plates at a density of 5 × 10^5^/ml and then subjected to a 24-h incubation with LPS. The JC-1 stain was added into the cell culture and incubated for 20 min in a CO_2_ incubator. Changes in red and green fluorescence were observed using a fluorescence microscope.

### Animals

Adult male C57BL/6 mice, weighing between 18 to 22 g, were procured from Zhejiang Vital River Laboratory Animal Technology Co., Ltd. (Zhejiang, China). These mice were housed in a facility that met the standard animal care requirements, where they were kept under a controlled environment with a consistent 12-h light and dark cycle, and provided with ad libitum access to food and water. All experimental protocols, including surgical procedures, were conducted in strict accordance with the ethical guidelines approved by the Animal Care and Use Committee of Tongji University School of Medicine. The study fully adhered to the recommendations outlined in the “Guide for the Care and Use of Laboratory Animals” published by the National Institutes of Health (NIH), ensuring the highest standards of animal welfare and ethical considerations throughout the research process.

### LPS-induced ARDS model and drugs treatment

The mice were systematically and randomly allocated into several experimental groups, each consisting of six mice. To simulate the ARDS condition in these mice, a lipopolysaccharide (LPS) solution was prepared by dissolving Escherichia coli strain 0111: B4 LPS, obtained from Sigma-Aldrich, headquartered in St. Louis, MO, USA, in sterile PBS. The LPS solution was then administered intraperitoneally to the test group mice at a dosage of 10 mg/kg body weight. In contrast, mice within the control group were treated with an equal volume of sterile PBS intraperitoneally, ensuring that any observed effects could be attributed specifically to the LPS exposure rather than the injection process itself. In the MT treatment group, MT treatment was given 30 min after LPS injection. Erastin (15 mg/kg; Selleck, USA) was dissolved in 5% dimethylsulfoxide (DMSO) and intraperitoneally injected three times every 12 h, and the last injection was 1 h before LPS treatment. Researchers were not blinded to the animal grouping.

### Bronchoalveolar lavage fluid (BALF) collection

Mice were anesthetized, the limbs were fixed, the neck was fully exposed, the skin of the neck was cut and the muscle was carefully separated, the trachea was exposed and punctured with an indwelling needle, the indwelling needle was fixed with a suture, a 1 ml syringe was connected and 0.8 ml of PBS was injected and repeatedly aspirated three times and collected.

### Measurement of protein concentration

The BALF samples were centrifuged at 800 × *g* for 5 min. The supernatant was collected and the protein content was determined using the BCA protein assay kit (Thermo Scientific, Rockford, USA).

### Alveolar macrophages isolation and purification

The collected BALF was centrifuged at 1200 rpm for 10 min, and the cells were washed with PBS and then inoculated with complete medium in 10 cm culture dishes for 1 h at 37 °C. The adherent cells were collected, suspended with PBS, incubated with surface antibodies (CD11c, F4/80) for 90 min at 4 °C, and subsequently detected by flow cytometry after suspension with PBS, and the proportion of each antibody positive was analyzed after labeling the CD11c and F4/80 positive clusters.

### Lung histopathology and lung injury score analysis

The lung tissues were subjected to fixation using a 4% paraformaldehyde solution for a duration of 24 h. Subsequently, the fixed lung tissues were embedded in paraffin following established protocols. The resulting paraffin blocks were then sliced into sections measuring 5 μm in thickness, which were subsequently stained with hematoxylin and eosin (HE). These sections were then examined under a microscope, and the extent of lung tissue damage was evaluated using precise criteria, including intra-alveolar hemorrhage, neutrophil infiltration in air spaces or vessel walls, and the thickness of the alveolar wall/hyaline membrane. Each criterion was allotted a score ranging from 0 to 4 on a graduated scale, where 0 represented mild and 4 signified severe injury. The cumulative total of these scores for each component constituted the overall lung injury score, with higher scores indicating increasingly severe damage.

### Immunohistochemical staining

Paraffin-fixed lung tissues were incubated at 37 °C overnight, then deparaffinized and treated with 3% H_2_O_2_ for 15 min. After microwave-based antigen retrieval and natural cooling for 40 min, the sections were blocked in 1% BSA for 30 min. Overnight incubation at 4 °C followed with a primary rabbit anti-4-HNE antibody (Bioss, China; 1:500 dilution). Finally, secondary fluorescent or biotin-labeled antibodies were applied to the tissues at 37 °C for 2 h, preceding microscope image capture.

### Statistical analysis

All results in the present study were presented as mean ± standard deviation (SD). Statistical analyses were performed using SPSS 17.0 software (SPSS, Chicago, USA) and GraphPad Prism 8.0 software. Student’s *t*-test was used to compare differences between two groups. Differences between multiple groups were compared by a one-way ANOVA analysis followed by Tukey’s post hoc test. Significance was considered at a level of *p* < 0.05.

### Supplementary information

Original Data File

Supplemental material

## Data Availability

Data will be made available on request.
