# Supplementary material for: Melatonin alleviates septic ARDS by inhibiting NCOA4-mediated ferritinophagy in alveolar macrophages
Source: Cell Death Discov. 2024 May 24;10:253. doi: 10.1038/s41420-024-01991-8 (PMC11126704; doi:10.1038/s41420-024-01991-8)

Original image：Fig 1 A

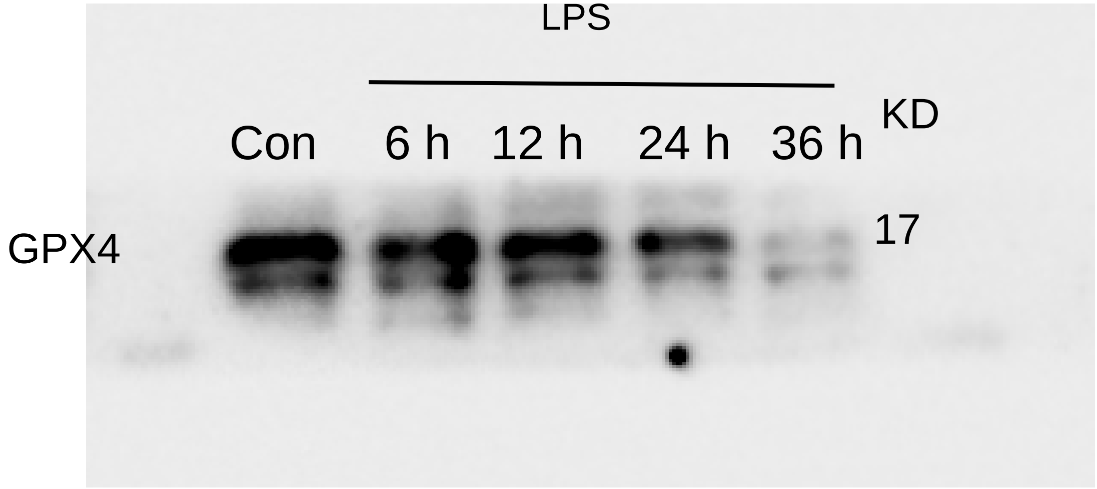

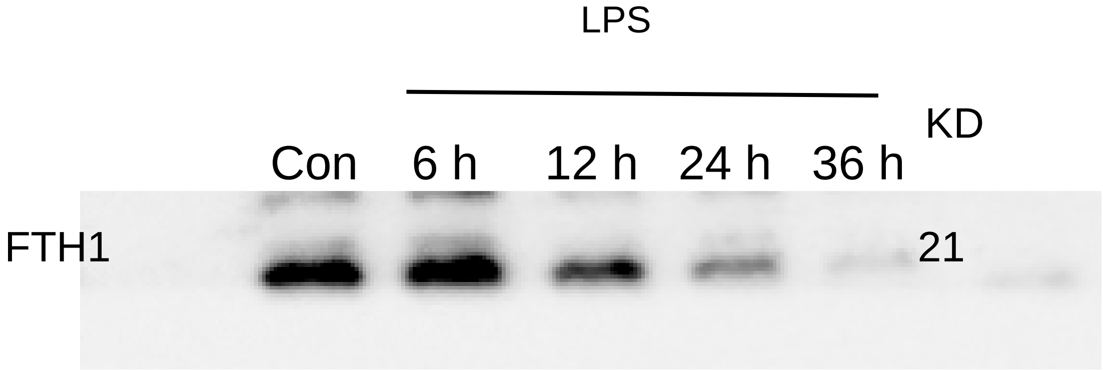

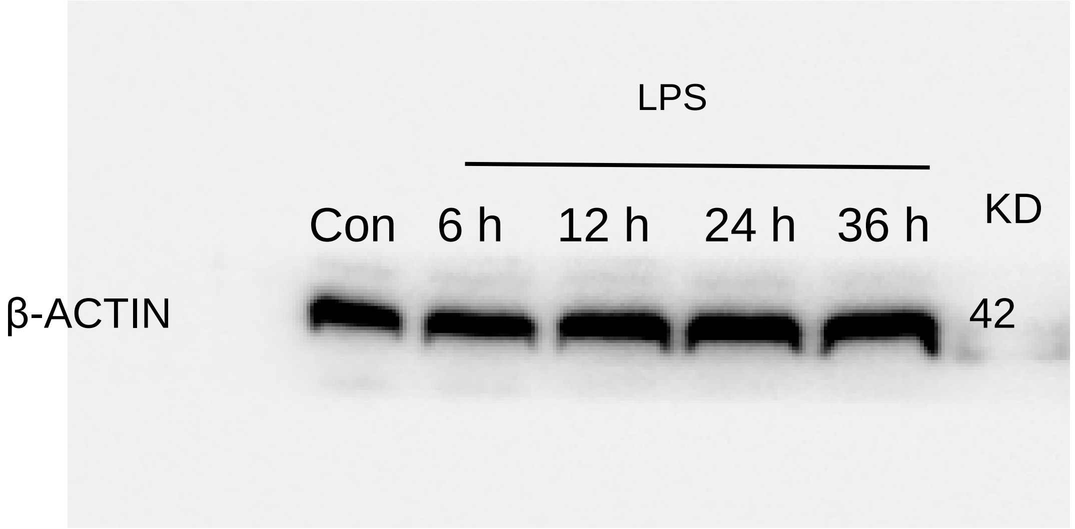

Original image：Fig 3 A

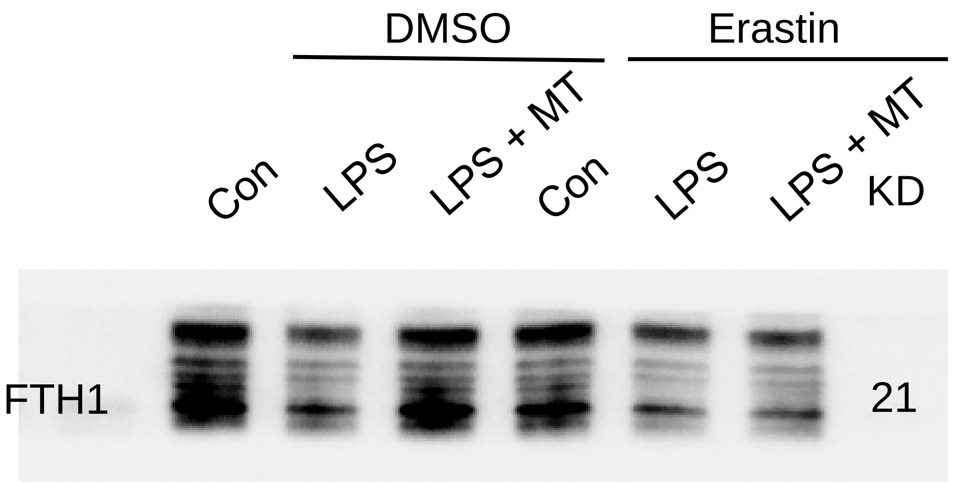

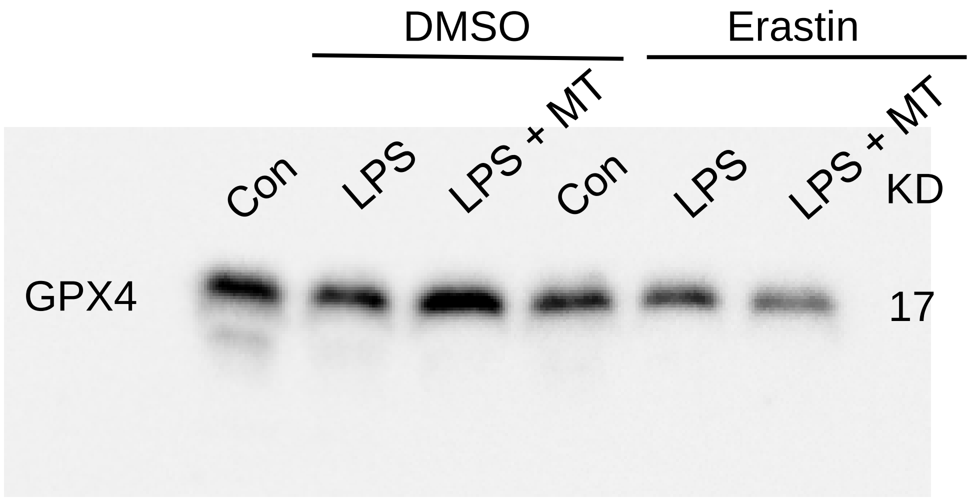

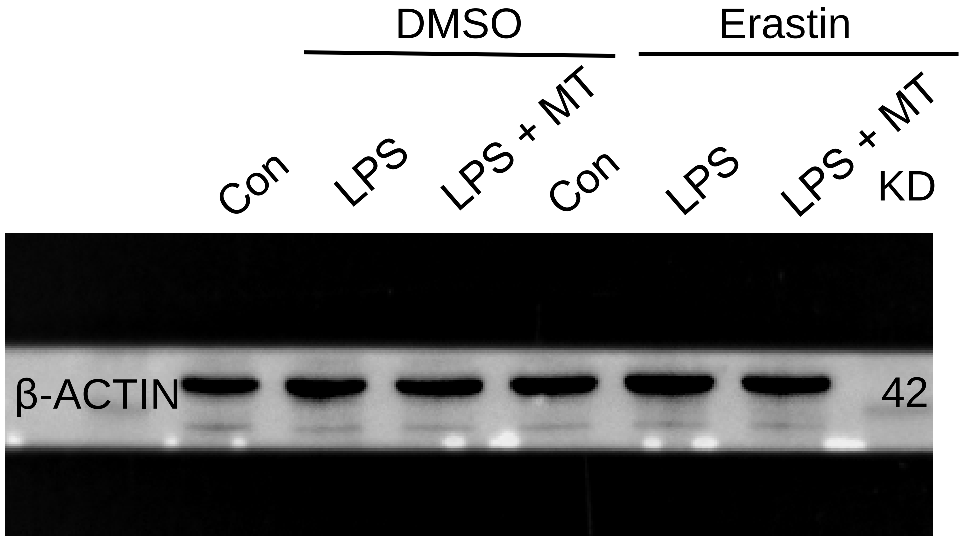

Original image：Fig 4 E

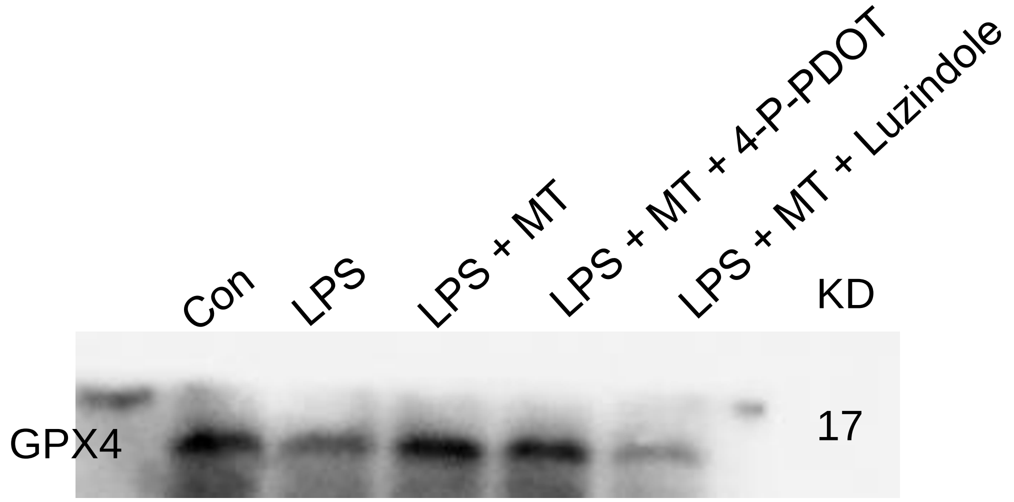

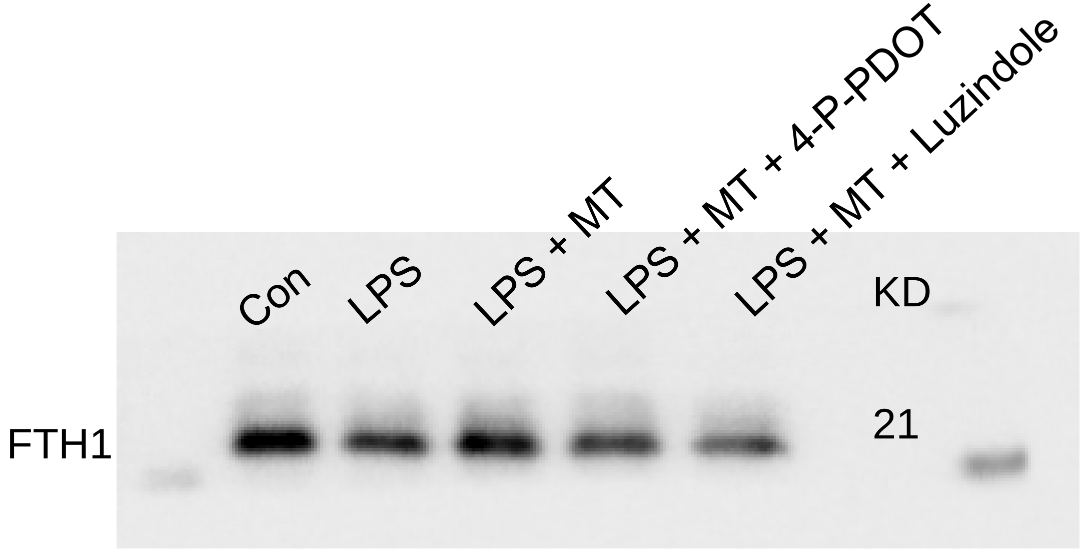

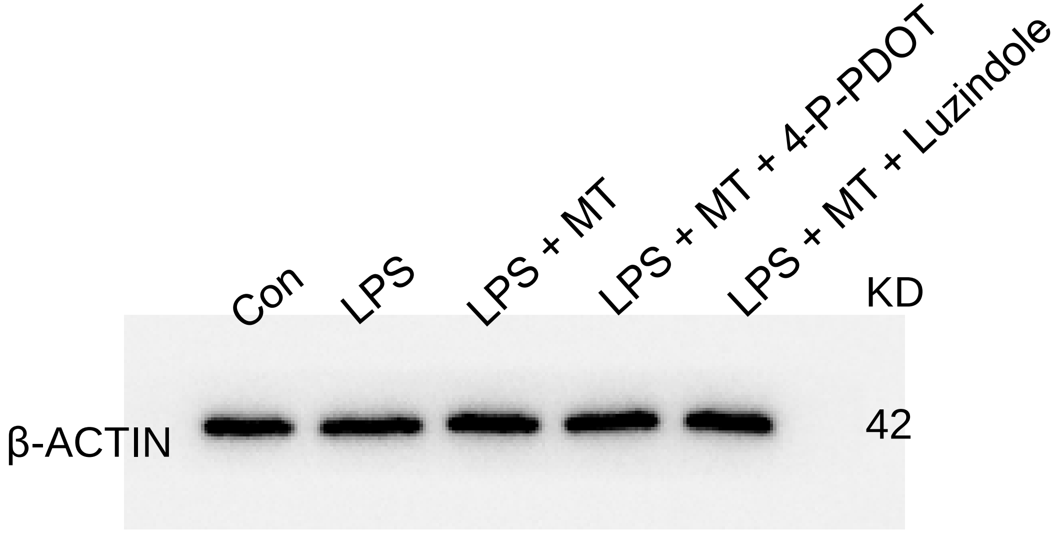

Original image：Fig 5 E

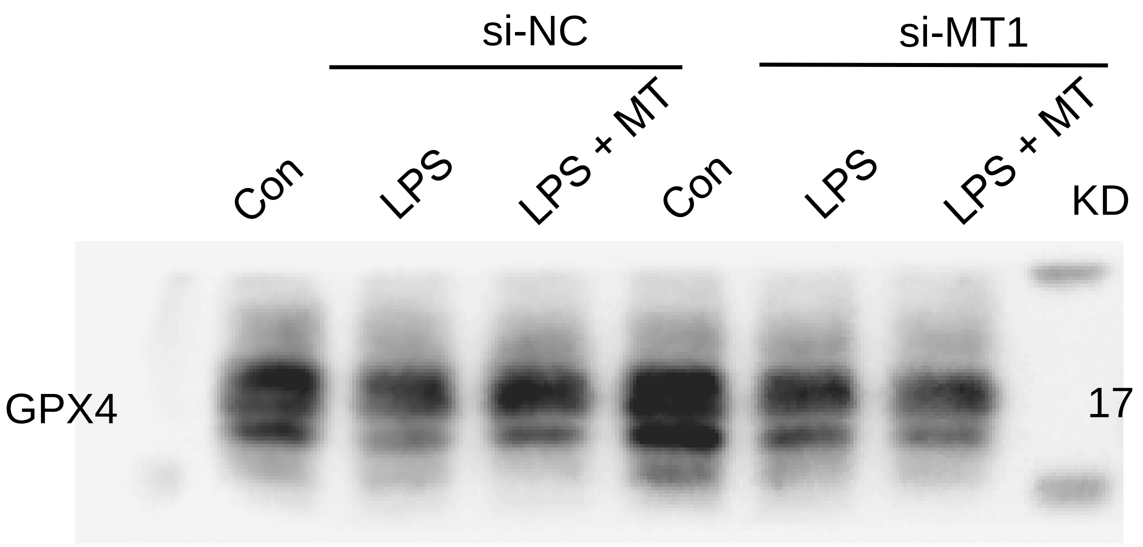

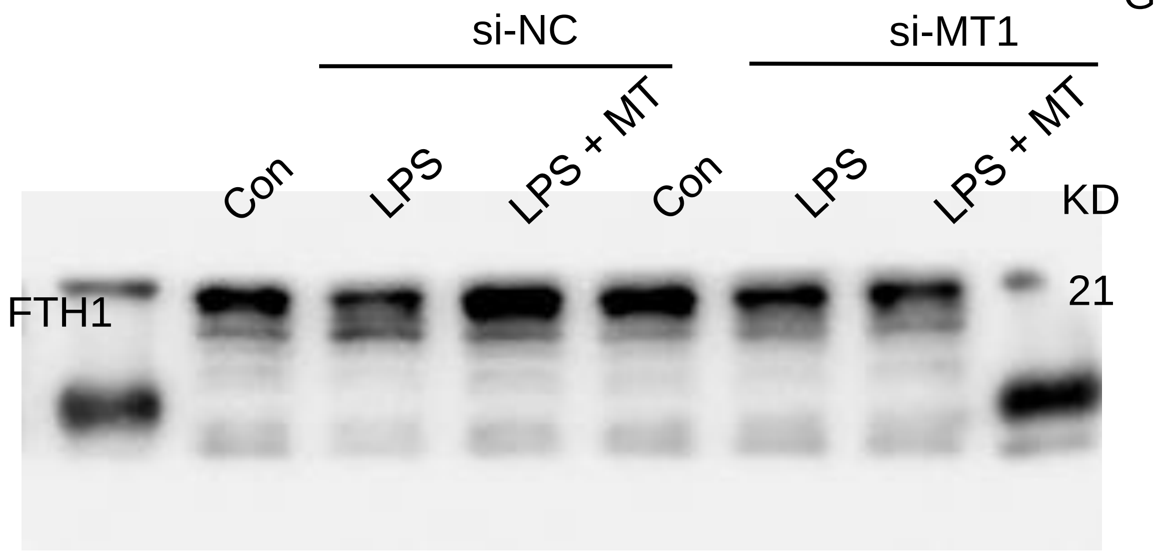

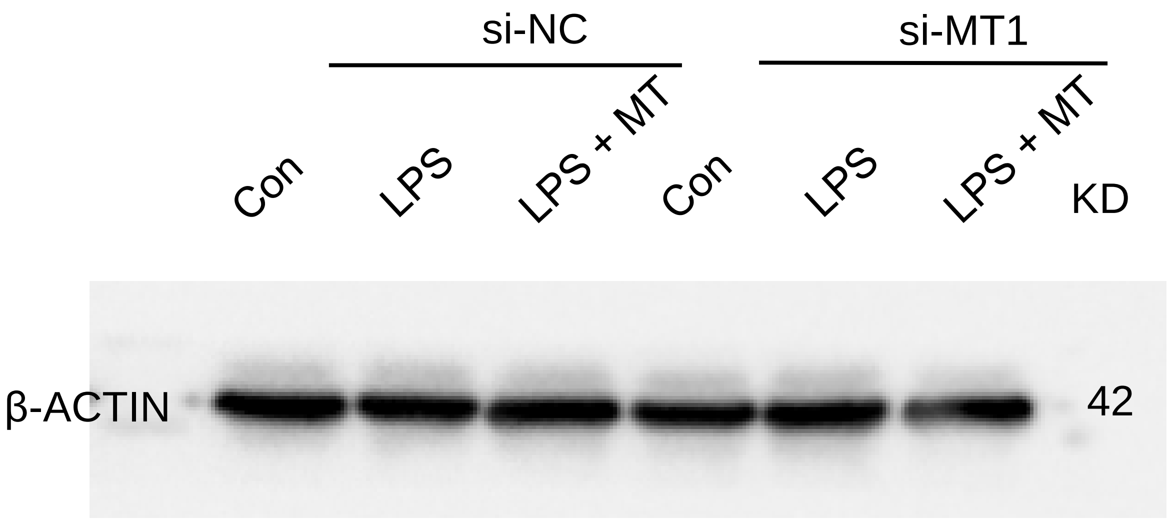

Original image：Fig 6 E

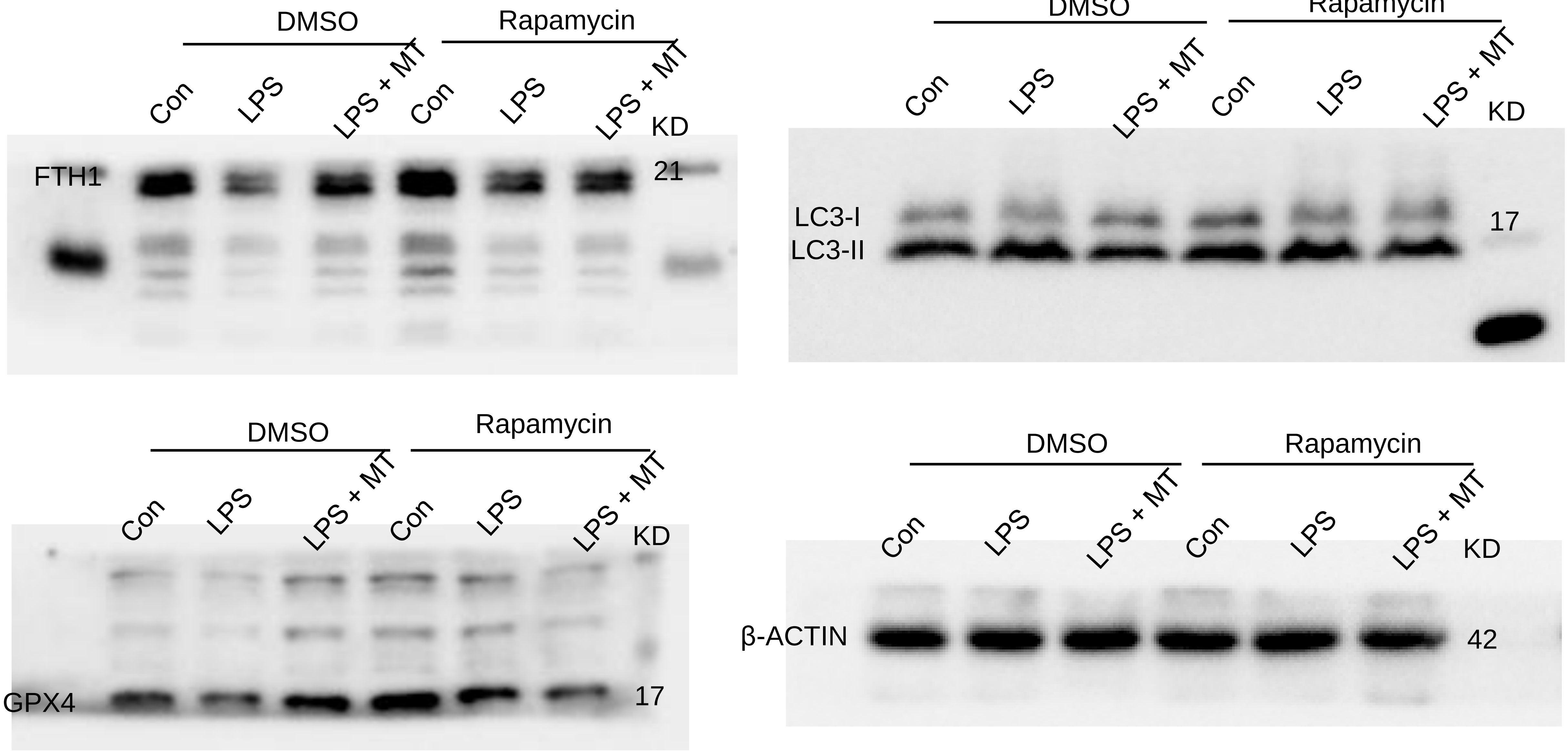

Original image：Fig 7 F

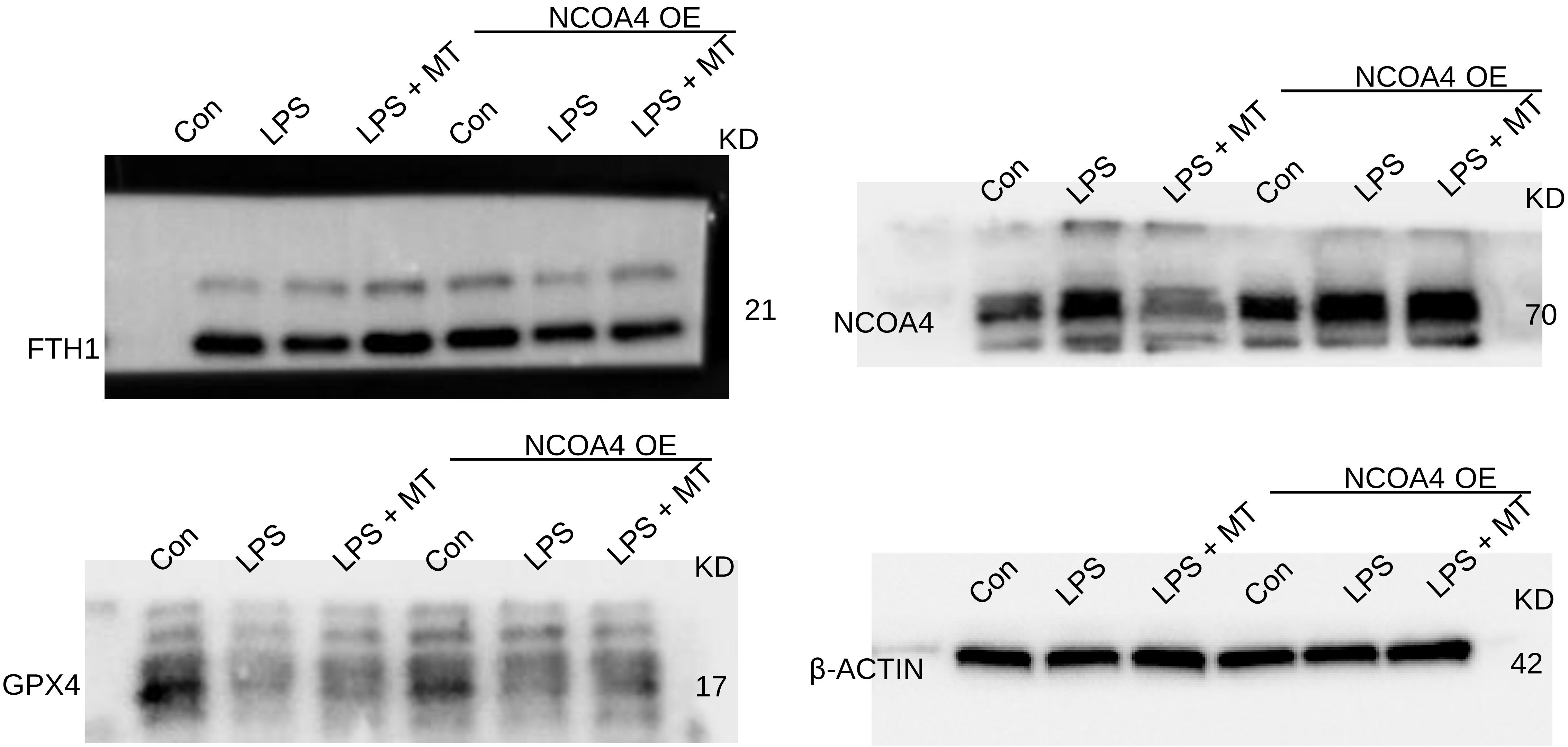

Original image：Fig 8 F

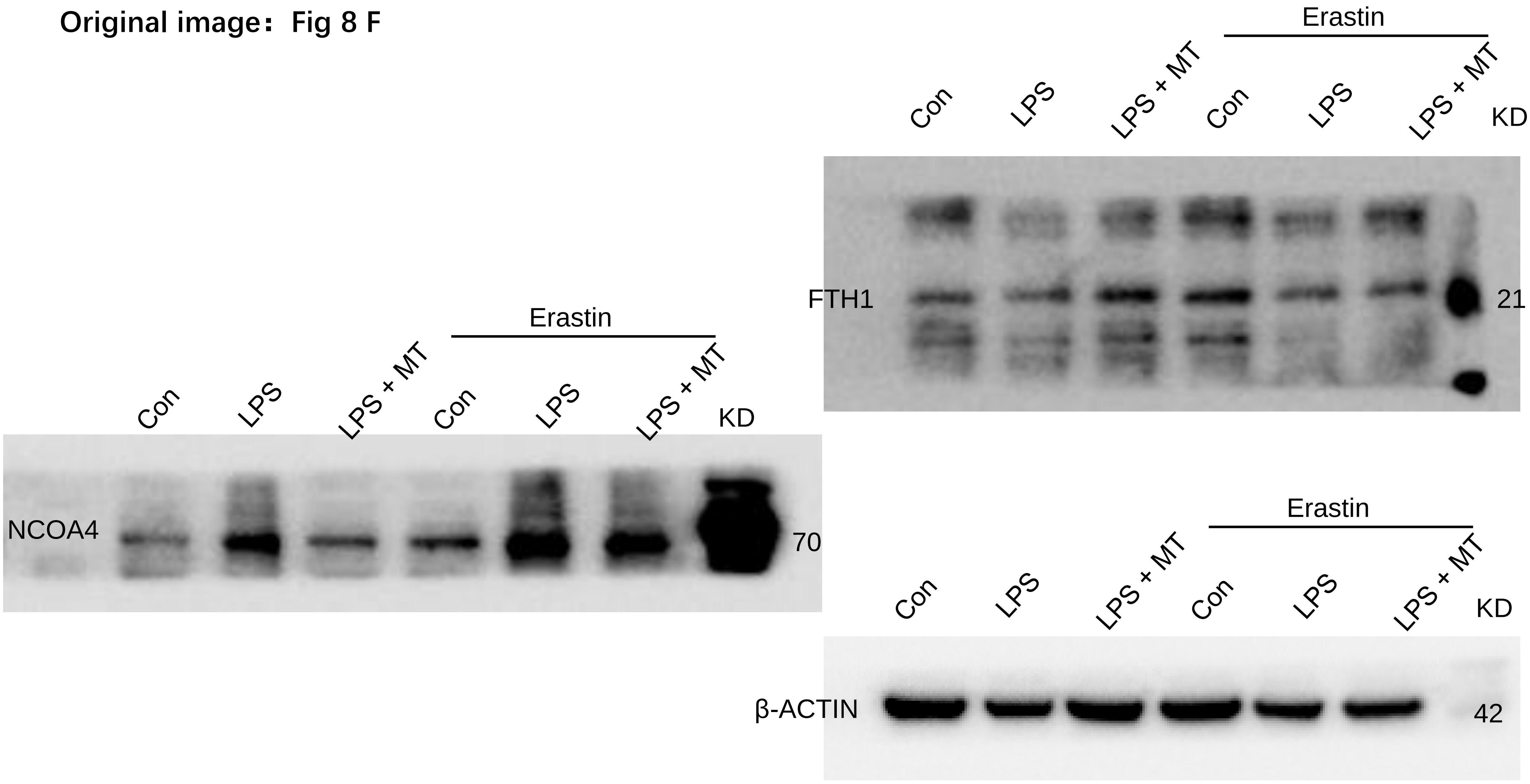

Supplement: Supplementary file 1 — Original Data File [file 41420_2024_1991_MOESM1_ESM.pdf]
