# Supplementary material for: Melatonin alleviates septic ARDS by inhibiting NCOA4-mediated ferritinophagy in alveolar macrophages
Source: Cell Death Discov. 2024 May 24;10:253. doi: 10.1038/s41420-024-01991-8 (PMC11126704; doi:10.1038/s41420-024-01991-8)
Supplement: Supplementary file 2 — Supplemental material [file 41420_2024_1991_MOESM2_ESM.docx]

**Supplementary**


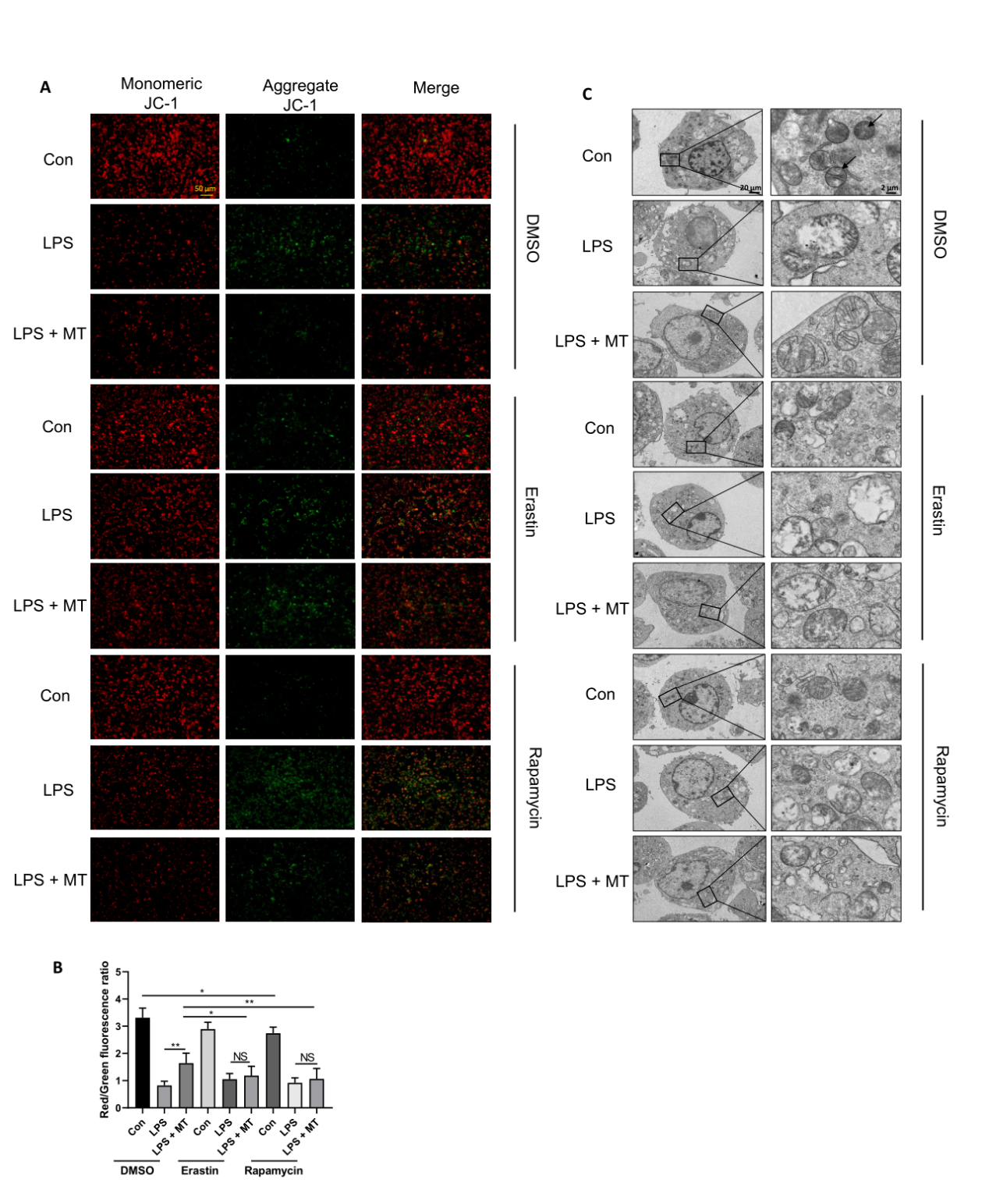


**Supplementary 1. MT attenuated mitochondrial damage in LPS-treated macrophages.** Mitochondrial membrane potential changes detected by JC-1 mitochondrial assay kit was observed under fluorescence microscope (A) and statistics as shown in Figure (B). (C) Mitochondrial morphological changes under electron microscopy (The arrows mark mitochondria). Data are expressed as mean ± SD, (n=6) * *p* < 0.05, ** *p* < 0.01 indicate significant differences from each group, NS indicates no significance.


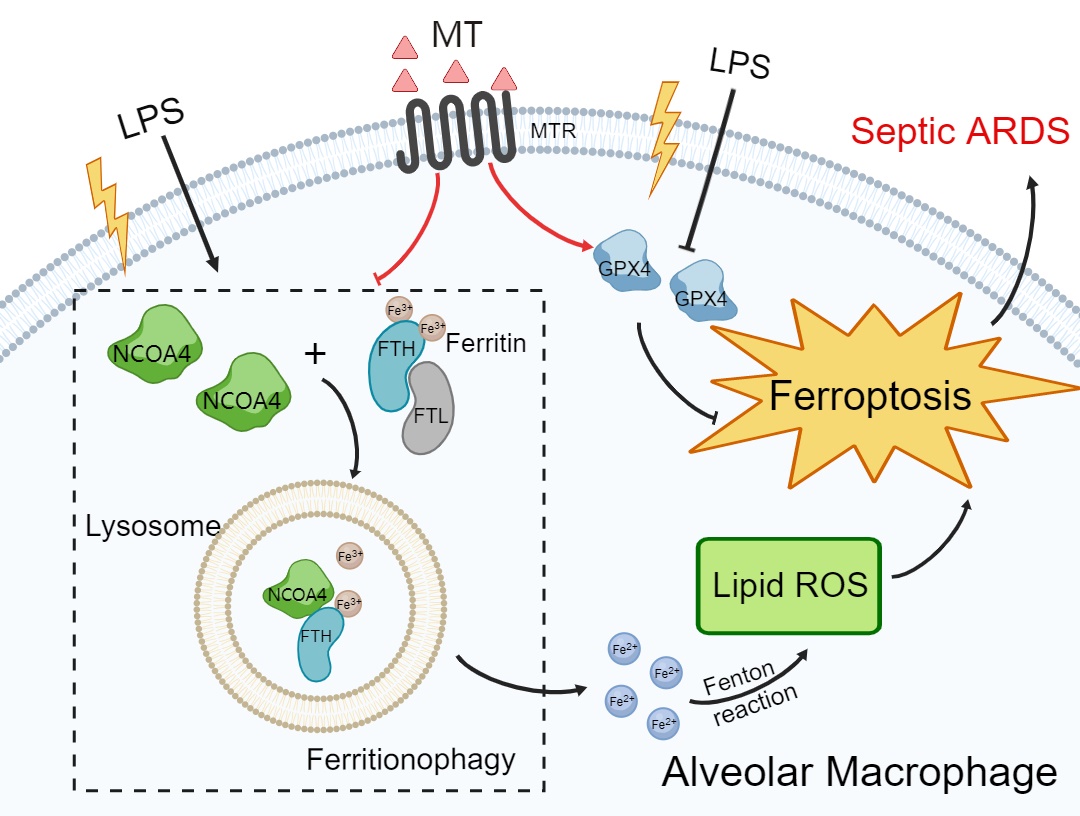


**Supplementary 2. Schematic representation of the putative mechanism by which MT suppresses ferroptosis by inhibiting NCOA4-induced ferritinophagy in AMs to alleviate septic ARDS.**
